# Descemet’s Membrane Supports Corneal Endothelial Cell Regeneration in Rabbits

**DOI:** 10.1038/s41598-017-07557-2

**Published:** 2017-08-01

**Authors:** Jingyao Chen, Zhiyuan Li, Liying Zhang, Shangkun Ou, Yanzi Wang, Xin He, Dulei Zou, Changkai Jia, Qianqian Hu, Shu Yang, Xian Li, Juan Li, Junqi Wang, Huimin Sun, Yongxiong Chen, Ying-Ting Zhu, Scheffer C. G. Tseng, Zuguo Liu, Wei Li

**Affiliations:** 1 Eye Institute of Xiamen University, Xiamen University Medical College, Xiamen, Fujian China; 2 Xiamen University affiliated Xiamen Eye Center, Xiamen, Fujian China; 3 Fujian Provincial Key Laboratory of Ophthalmology and Visual Science, Xiamen, Fujian China; 4 Yan’an Hospital of Kunming Medical University, Kunming, Yunnan China; 5 The Affiliated Hospital of Southern Medicine University in Chenzhou, Chenzhou, Hunan China; 6 Ocular Surface Center, Miami, Florida USA; 7 Shandong Eye Hospital, Shandong Eye Institute, Shandong Academy of Medical Sciences, Jinan, Shandong China

## Abstract

Descemet’s membrane (DM) helps maintain phenotype and function of corneal endothelial cells under physiological conditions, while little is known about the function of DM in corneal endothelial wound healing process. In the current study, we performed *in vivo* rabbit corneal endothelial cell (CEC) injury via CEC scraping, in which DM remained intact after CECs removal, or via DM stripping, in which DM was removed together with CECs. We found rabbit corneas in the CEC scraping group healed with transparency restoration, while there was posterior fibrosis tissue formation in the corneas after DM stripping on day 14. Following CEC scraping on day 3, cells that had migrated toward the central cornea underwent a transient fibrotic endothelial-mesenchymal transition (EMT) which was reversed back to an endothelial phenotype on day 14. However, in the corneas injured via DM stripping, most of the cells in the posterior fibrosis tissue did not originate from the corneal endothelium, and they maintained fibroblastic phenotype on day 14. We concluded that corneal endothelial wound healing in rabbits has different outcomes depending upon the presence or absence of Descemet’s membrane. Descemet’s membrane supports corneal endothelial cell regeneration in rabbits after endothelial injury.

## Introduction

Corneal endothelial cells (CECs) arise from the neural crest and form a monolayer of hexagonal cells in the posterior surface of the cornea^1^. They act as a barrier between the corneal stroma and the aqueous humor, and regulate stromal hydration through the barrier and pump functions, thus playing a critical role in the maintenance of corneal transparency. Corneal endothelial cells in cats^2, 3^, monkeys^4^, pigs^5^, and humans^6, 7^ display limited proliferative capacity *in vivo*; severe endothelial damage or dystrophy in these species will compromise the function of corneal endothelium and cause vision loss. In humans, endothelial cell damage due to mechanical injury, such as that occurring during intraocular surgery, has been a major cause of corneal opacification, leading to endothelial transplantation^8^. However, CECs in other species, such as rat^9^ and rabbit^2^ can proliferate *in vivo*. Among these species, rabbits are extensively used as experimental models to investigate the cellular biology of corneal endothelial wound healing and provide much insight into this process.

Currently, mechanical scrape injury^10^ and the transcorneal freeze injury^2, 11^ models are commonly used to characterize endothelial wound healing biology. Following a scrape injury in a rabbit, cells at the leading edge of the wound spread and slide into the denuded area^12, 13^. The wound healing process includes CEC migration, elongation, coalescence, and mitosis procedures^14^. Despite these behavioral changes, the endothelial cells maintain a normal phenotype^10^. However, endothelial wound healing in a rabbit after a freeze injury results in retrocorneal fibrous membrane formation and corneal transparency declines^10, 15^. A similar change was also found after alkali injuries in rabbits^16^ and in humans^16–21^. It was suggested that this corneal endothelial fibrogenic response is caused by endothelial mesenchymal transition (EMT) activation leading to myofibroblast transdifferentiation^22, 23^. In these two endothelial injury models, Descemet’s membrane (DM) maintained intact^10^. However, DM injury is commonly encountered along with endothelial damage after intraocular surgery and ocular trauma^24–28^. Therefore, the relevance of these models to conditions encountered in clinical practice is limited. In fact, till now little is known about the contribution made by the DM to corneal endothelial wound healing.

DM is a specialized basement membrane of the corneal endothelium anchoring the corneal endothelium and provides a stable scaffold during morphogenesis^29^. It is mainly composed of collagen III and VIII and glycoproteins organized into a 3-dimensional filamentous network^30^. DM is synthesized by corneal endothelial cells during both the prenatal and postnatal periods of life^31^, and thickens with age^32^. It contributes to maintaining the CEC phenotype and function under physiological conditions^33, 34^. In this study, we compared the rabbit corneal endothelial wound healing responses induced by DM stripping and the CEC scraping method to determine the contribution made by the DM to restoring corneal deturgescence and transparency. Our results indicate that DM indeed plays a critical role in corneal endothelial cell regeneration and function restoration in rabbits.

## Results

### Corneal endothelial wounding model

**Figure 1:**
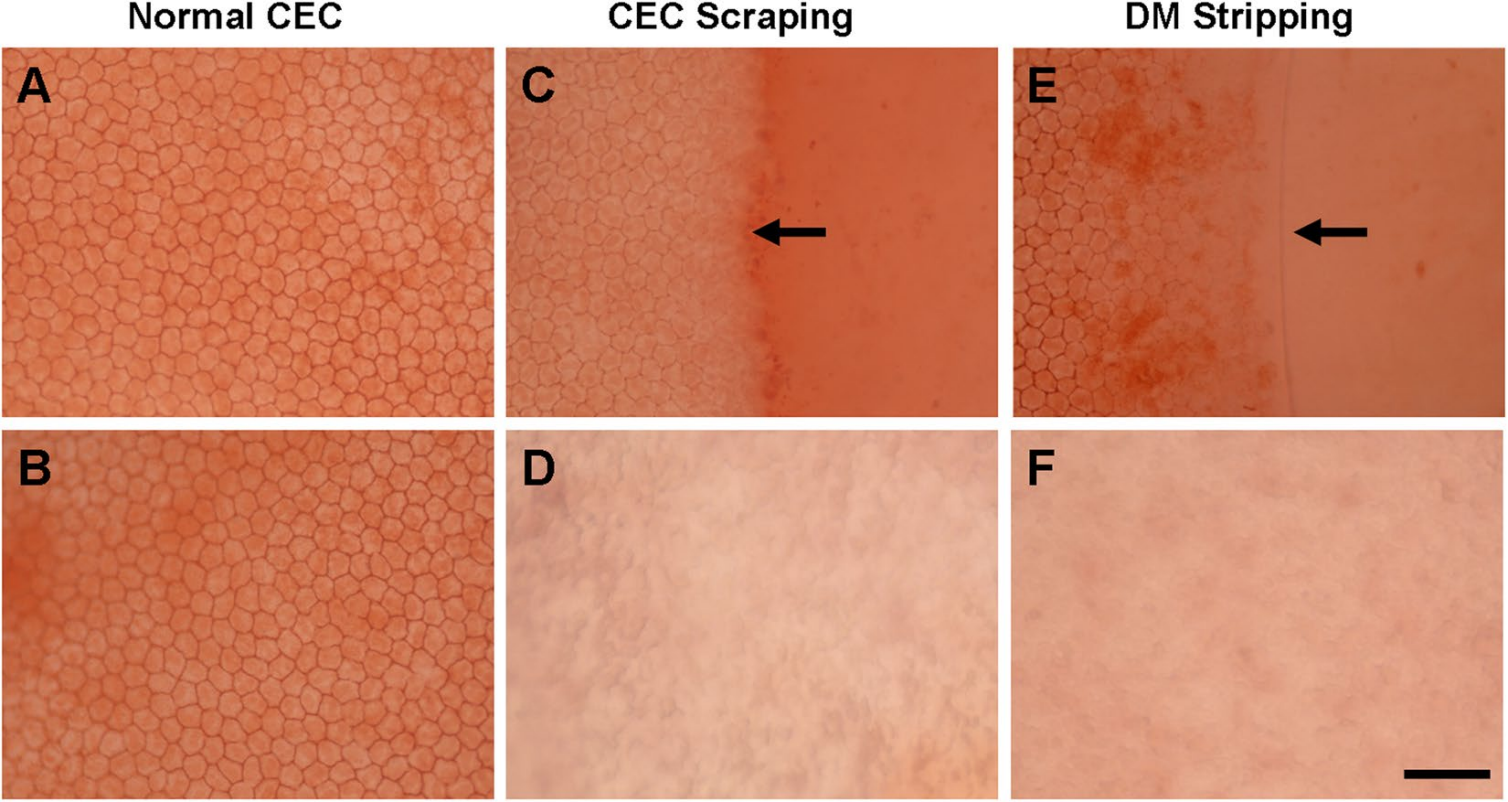
Alizarin red S staining of corneal endothelium. Alizarin red S staining showed endothelial cells in peripheral (**A**) and central (**B**) region of the normal rabbit cornea. In the CEC scraping group, cell damage border (arrow) could be seen just after the injury (**C**). Within the scraping area, there was no endothelial cell (**D**). In the DM stripping group, the cutting edge of the DM (arrow) was visible (**E**), and there was no endothelial cell inside the DM cutting edge (**E**) and the central cornea after DM stripping (**F**). Bar represents 100 μm.

We used Alizarin red S staining of the corneal whole-mount to demonstrate the validity of the corneal endothelial wounding model. Normal rabbit corneal endothelial cells from both peripheral (Fig. 1A) and central (Fig. 1B) corneal region showed intact cell sheet with hexagonal cell shape. In the CEC scraping group, Alizarin red S staining revealed clear cell damage border (Fig. 1C). Within the scraping area, there was no endothelial cell staining (Fig. 1D). In the DM stripping group, the stripping line of the DM was visible under the microscope (Fig. 1E). Endothelial cells outside the stripping line remained the regular shape, while there was no cell present inside the stripping line, as well as the central cornea after DM stripping (Fig. 1F). These results proved that both CEC scraping and DM stripping method successfully created corneal endothelial wounding model, and there was no endothelial cell remained in the wounding area.

### Corneal appearance after endothelial damage

**Figure 2:**
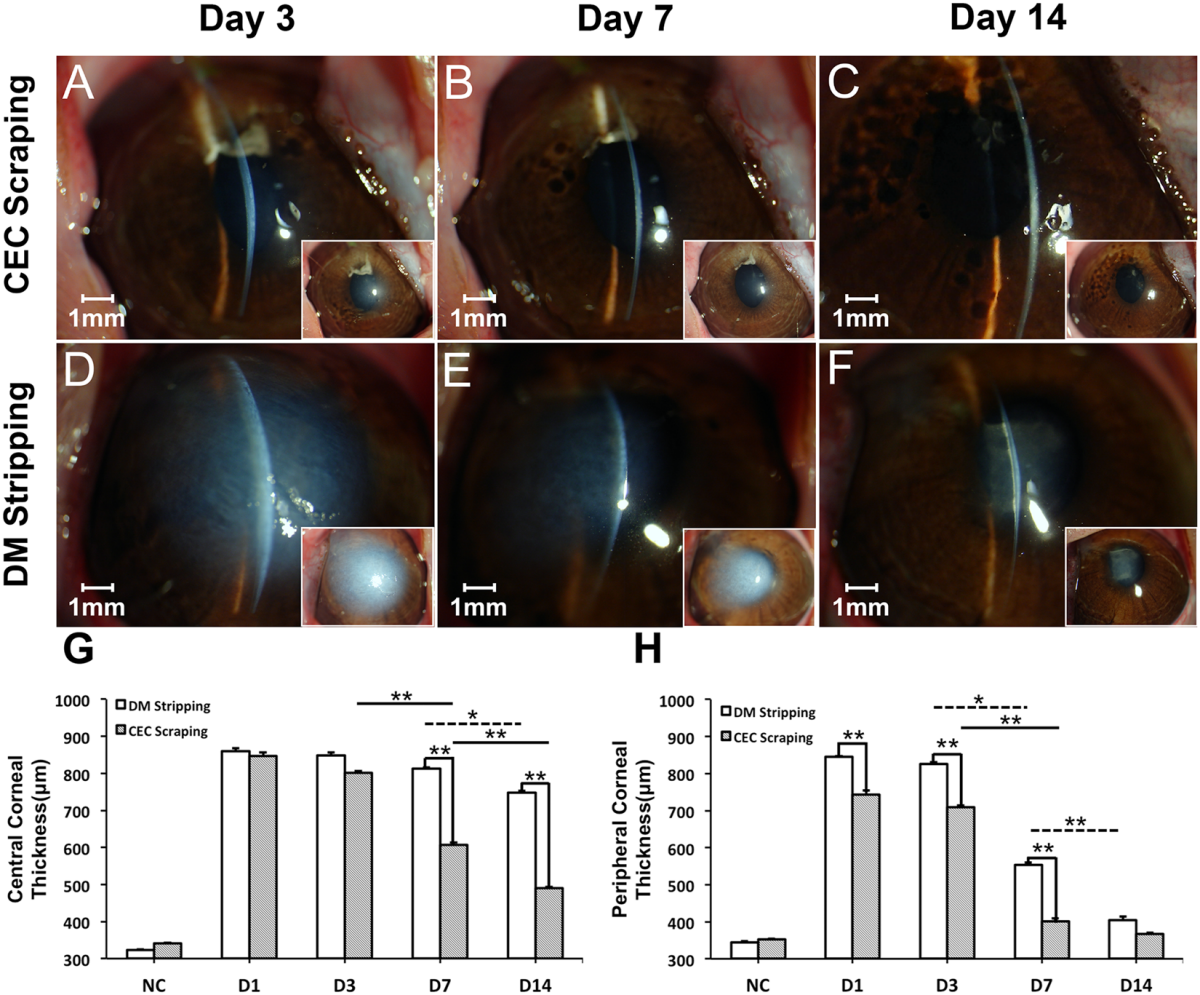
Slit-lamp microscopy observation and corneal thickness change after CEC injury. Slit-lamp microscope image of rabbit cornea after CEC scraping injury (**A**,**B**,**C**) or DM stripping injury (**D**,**E**,**F**) for different durations. Both central corneal thickness (**G**) and peripheral corneal thickness (**H**) dramatically increased one day after the endothelial injury (D1), compared with that of the normal control before injury (NC). After that, there was gradual decrease of central and peripheral corneal thickness in both groups from day 3 to day 14 (*p < 0.05; **p < 0.01). Central corneal thickness was higher in DM stripping group at day 7 and day 14 (**p < 0.01), while peripheral corneal thickness was higher in DM stripping group at day 1, day 3, and day 7 (**p < 0.05).

The rabbit corneas after CEC scraping showed moderate stromal edema of the surgical site on day 3 (Fig. 2A), and then decreased on day 7 (Fig. 2B). On day 14, the corneas returned to a normal appearance with transparent corneal stroma (Fig. 2C). In contrast, the rabbit corneas after DM stripping showed severe stromal edema in both the central corneas and the peripheral corneas on day 3 (Fig. 2D). There was obvious reduction of corneal edema on day 7 (Fig. 2E) and complete peripheral corneal edema release on day 14 (Fig. 2F). However, there was posterior fibrosis tissue formation in the surgical site on day 14, while the anterior parts of the stroma remained transparent under the slit-lamp microscope (Fig. 2F). The corneal thicknesses in the injury area and peripheral cornea were measured by Ultrasonic A pachymeter. The results showed that both CEC scraping and DM stripping induced dramatic corneal edema in the injury area 24 hours post-surgery. There was only a slight central corneal thickness decrease from day 1 to day 7, and a significant decrease from day 7 to day 14 in the rabbits after DM stripping, while the central corneal thickness gradually decreased from day 3 to day 14 in the rabbits after CEC scraping (Fig. 2G). Regarding the peripheral cornea, both CEC scraping and DM stripping induced a significant increase of the thickness on day 1, and the thickness was maintained at a high level on day 3, significantly decreased on day 7, and returned to normal level 14 days post-surgery (Fig. 2H). The peripheral cornea was thicker in DM stripping group compared with that of the CEC scraping group on days 1, 3, and 7 (Fig. 2H).

### CECs regeneration during wound healing

**Figure 3:**
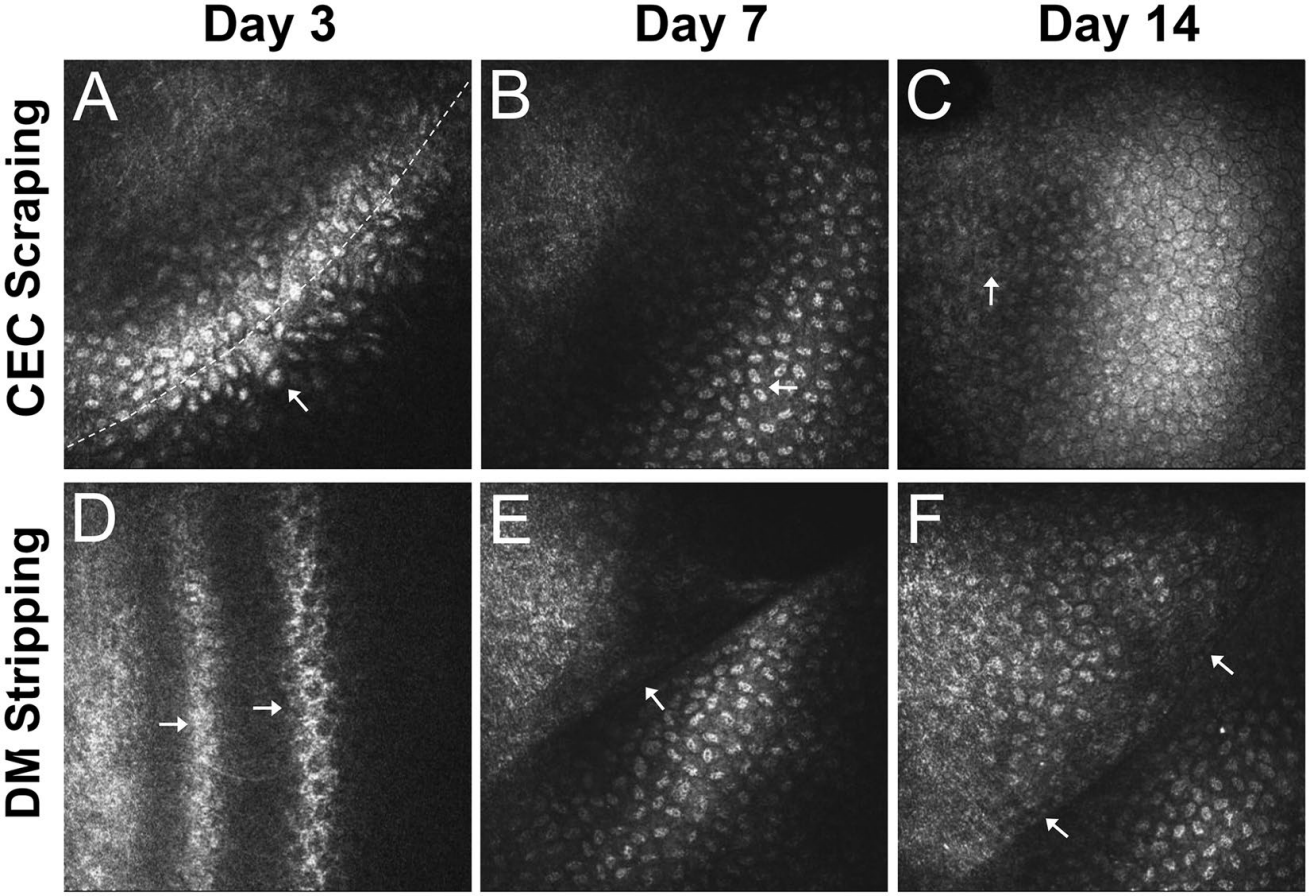
*In vitro* confocal microscopy image of CECs during wound healing. In the CEC scraping rabbits, endothelial cells around the surgical boundary (dotted line) changed to an elongated shape (arrow) on day 3 (**A**), became flatter and smaller with high nucleus reflection (arrow) on day 7 (**B**), and returned to the typical hexagonal mosaic appearance with visible cell border (arrow) on day 14 (**C**). In the DM stripping rabbits, CECs could not be well distinguished on day 3 (**D**). On day 7, the surgical boundary became clear (arrow), and there were very few cells presented inside the DM stripping area, which is on the upper-left side of the arrow (**E**). Cell density inside the DM stripping area (upper-left side of the arrows) dramatically increased, and some cells showed an atypical hexagonal shape on day 14 (**F**).

*In vitro* confocal microscopy was performed on the corneas to observe in detail the morphological changes of the CECs during wound healing. In the CEC scraping rabbits, the endothelial cells around the surgical boundary area showed increased cell volume and high nuclear reflection on day 3; the otherwise clear endothelial cell border disappeared; endothelial cell morphology changed from the typical hexagonal into an elongated fusiform, which pointed to the damaged area; and there were no cells present in the central part of the scraping area (Fig. 3A). On day 7, the CECs continued to move toward the center area, cells became flatter and smaller with high nucleus reflection, the cell border remained unclear, and density of the forefront migrating cells was low (Fig. 3B). On day 14, cells already covered the entire surgical site. Cells in the para-central surgery region returned to the typical hexagonal mosaic appearance and the cell borders were clearly visible (Fig. 3C). In the DM stripping rabbits, there was severe stromal edema of the surgical area, and the endothelial cells could not be well distinguished under confocal microscope on day 3 (Fig. 3D). On day 7, the surgical boundary became clear, endothelial cells outside the stripping line showed heterogeneous morphology and indistinct borders, and there were only few cells inside the stripping line (Fig. 3E). On day 14, cells outside the stripping line still had not recovered the hexagonal shape, while the cell density inside the stripping line dramatically increased, and some cells showed an atypical hexagonal shape (Fig. 3F).

### Histological changes of the cornea after CEC injury

**Figure 4:**
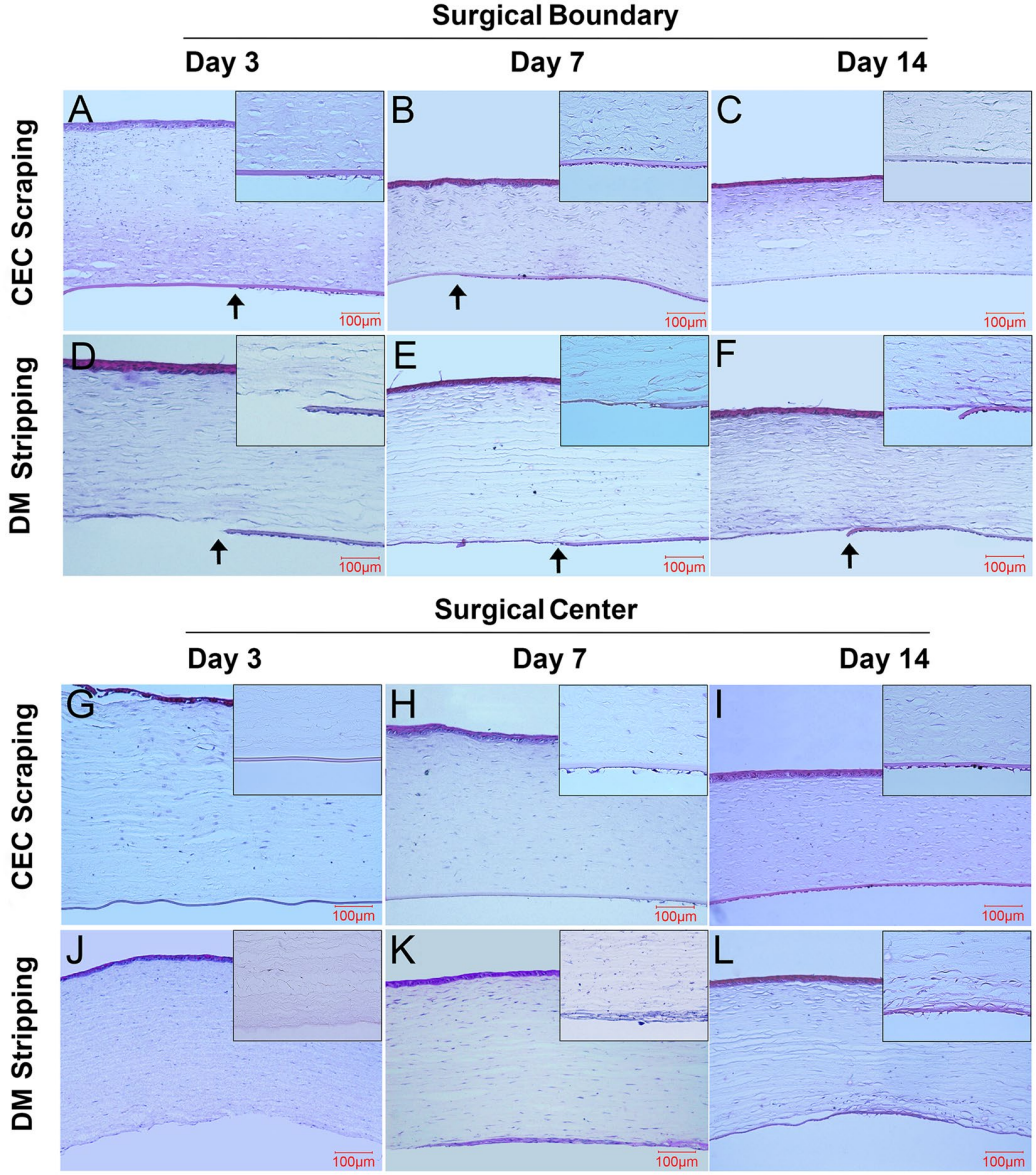
H&E staining of the corneal tissues after endothelial injury. The DM was intact in the CEC scraping group, and a leading edge of CECs was detectable on day 3 (**A**, arrow) and day 7 (**B**, arrow) but vanished on day 14 (**C**). The DM stripping cornea showed a clear border of DM removal on day 3 (**D**, arrow), day 7 (**E**, arrow), and day 14 (**F**, arrow). In the central cornea, the DM was denuded on day 3 in CEC scraping rabbits (**G**), and there were few cells on the DM surface on day 7 (**H**), while the DM was covered by cells on day 14 (**I**). In the DM stripping rabbits, the central cornea showed a rough surface without the DM on day 3 (**L**) and was covered with a membranous tissue on day 7 (**M**), which remained on day 14 (**N**). Bars represent 100 μm.

We harvested the corneal tissue at different time points and performed H&E staining on the tissue cryosections. On the surgical boundary of the CEC scraping group, the DM was intact, and the CECs on the surgical border showed loose structure and low cell density on day 3 (Fig. 4A). The CECs migrated toward the corneal center on day 7 (Fig. 4B), and the CECs’ density on the surgical boundary returned to a normal stage on day 14 (Fig. 4C). The DM stripping corneas showed clear borders of DM removal and exposed posterior corneal stroma on day 3 (Fig. 4D). On day 7, there were cells covering some parts of the stripping area (Fig. 4E), and the density increased on day 14 (Fig. 4F).

In the CEC scraping rabbits, there were no cells present on the surface of the DM in the central cornea area 3 days post-injury (Fig. 4G). On day 7, a few cells had migrated to the central region with loose structure and low density (Fig. 4H), while there were cells covering the entire center corneal posterior surface on day 14 (Fig. 4I). In the DM stripping rabbits, the central corneas showed a rough surface without DM, and there were few nuclei present on the stromal surface on day 3 (Fig. 4J). On day 7, a membranous tissue with cells covered the central DM stripping area with higher cellularity than other part of the stroma (Fig. 4K), and the membrane became more condensed on day 14 (Fig. 4L).

### Potential cell source of regenerated endothelial cells during wound healing

**Figure 5:**
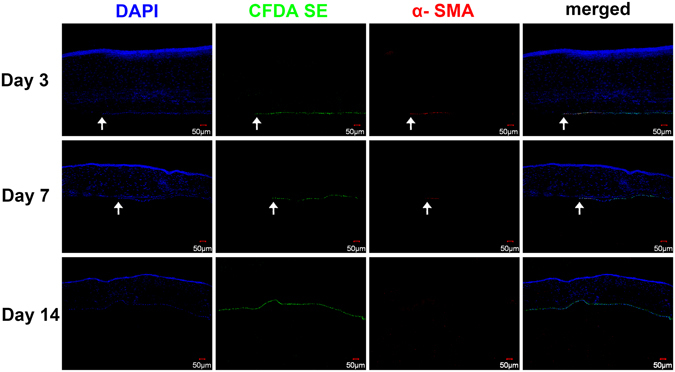
Endothelial cell tracing and α-SMA staining of cornea after CEC scraping. CFDA SE-labeled cells shows the leading edge of CEC injury (arrow) on day 3 and day 7, and complete CEC healing on day 14. Immunostaining showed that CFDA SE-labeled cells close to the leading edge were strongly expressed with α-SMA on day 3 (arrow), decreased on day 7 (arrow), and became negative on day 14. Bars represent 50 μm.

To determine the cell source and phenotype of these migrating cells during the corneal endothelial wound healing process, corneal endothelial cells were tagged with CFDA SE Cell Tracer 24 hours before the surgery. After CEC scraping, the non-scraped area showed green fluorescence on the posterior surface of the cornea, stands for the pre-labelled endothelial cells. The labeled cells migrated toward the central cornea on day 7 and covered the entire surface on day 14, indicating complete healing of the endothelial wound (Fig. 5). Immunofluorescence staining of α-SMA showed that the CFDA SE-labeled cells close to the wound edge were strongly expressed with α-SMA on day 3 and then decreased on day 7. After CEC wound closure on day 14, α-SMA became negative (Fig. 5).

**Figure 6:**
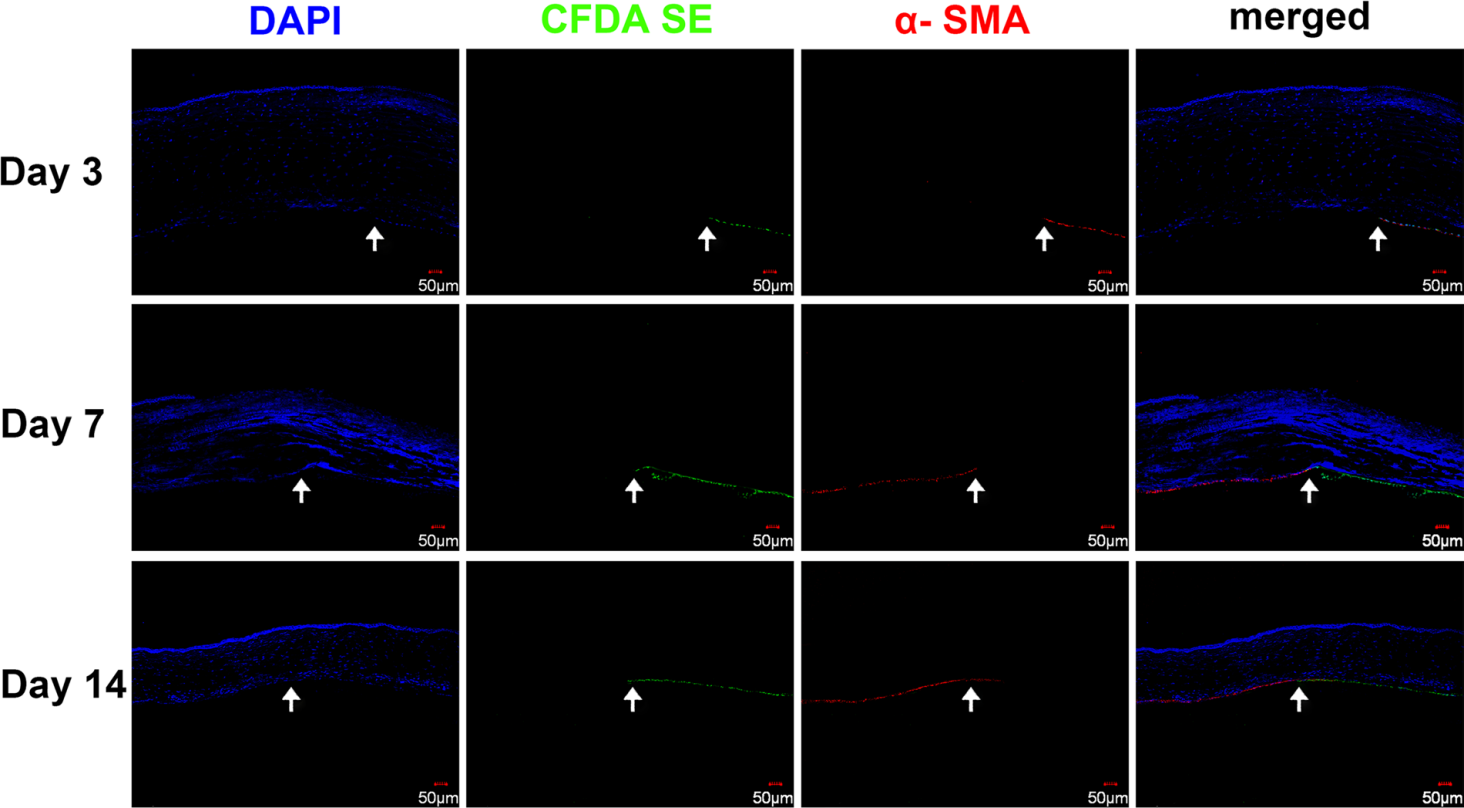
Endothelial cell tracing and α-SMA staining of the cornea after DM stripping. CFDA SE-labeled cells showed minor migration toward the central cornea from day 3 to day 14. Immunostaining showed that some CFDA SE-labeled cells close to the wound edge (arrows) expressed α-SMA on day 3. On day 7, cells on the stromal surface of the surgical site showed positive α-SMA staining, but they were not labeled with CFDA SE. CFDA SE-labeled α-SMA positive cells became negative on day 7. Cells on the stromal surface of the surgical site remained positive α-SMA staining on day 7 and day 14. Bars represent 50 μm.

In the DM stripping rabbits, absence of CFDA SE green fluorescence clearly demonstrated the endothelial wound area (Fig. 6). Although there was migration of CFDA SE-labeled cells toward the central cornea on day 7, there remained in some areas a lack of green fluorescence on day 14, indicating that this area was not covered by endothelial cells. Immunostaining showed that some CFDA SE-labelled cells close to the wound edge expressed α-SMA on postoperative day 3. On day 7, cells on the stromal surface of the surgical site showed strong α-SMA staining; however, these cells were not labelled with CFDA SE, indicating that they did not originate from corneal endothelial cells. Moreover, the previously α-SMA positive CFDA SE-labelled cells became α-SMA negative on day 7. On day 14, undamaged CECs located away from the surgical border remained α-SMA negative, while cells on the stroma surface of the surgical site showed positive α-SMA staining, and the majority of these cells were not labelled with CFDA SE (Fig. 6). These results indicated that, rather than some migrated endothelial cells outside the surgical site, majority of the cells covered the wound area after DM stripping were from other cell source instead of the corneal endothelial cells.

### CEC phenotype transition during wound healing

**Figure 7:**
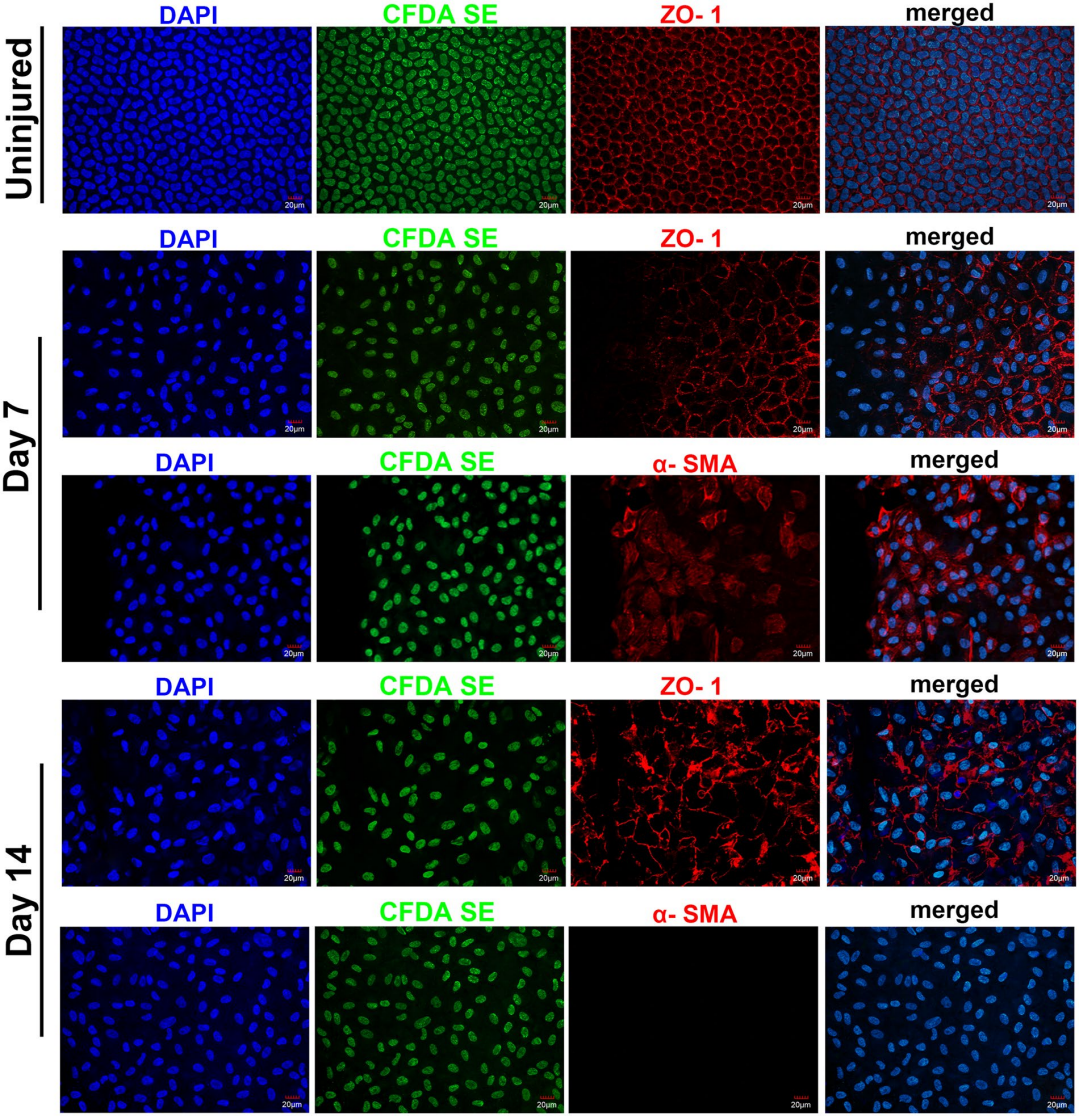
Whole-mount staining of ZO-1 and α-SMA on a CEC scraping cornea. ZO-1 expression decreased in the forefront of the migrating cells, while α-SMA was expressed in the migrating CECs on day 7. At day 14, ZO-1 was re-expressed on the cell border, although it was not as organized as that in the normal endothelium, while α-SMA became negative in all the endothelial cells. Bars represent 20 μm.

In order to further determine the CECs’ phenotype and function in the process of wound repair, whole-mount immunofluorescence staining of α-SMA and zonula occludens-1 (ZO-1) were performed. The ZO-1 expression in normal corneal endothelium showed integral structure with a clear boundary (Fig. 7). In the rabbits to which scraping injuries were administered, CECs centripetally migrated near the surgical area. The ZO-1 turned sparse and irregular, and even entirely disappeared in the forefront of the migrating cells. Meanwhile, α-SMA was expressed in the migrating CECs, especially in the forefront of the cells on day 7. At the late stage of the wound healing process of day 14, CECs resurfaced the surgical area, but the cell density was obviously much lower than that of the normal cornea before injury. The ZO-1 was re-expressed on the cell border, although it was not as organized as that in the normal endothelium. Interestingly, α-SMA became negative in all the endothelial cells on day 14 (Fig. 7).

**Figure 8:**
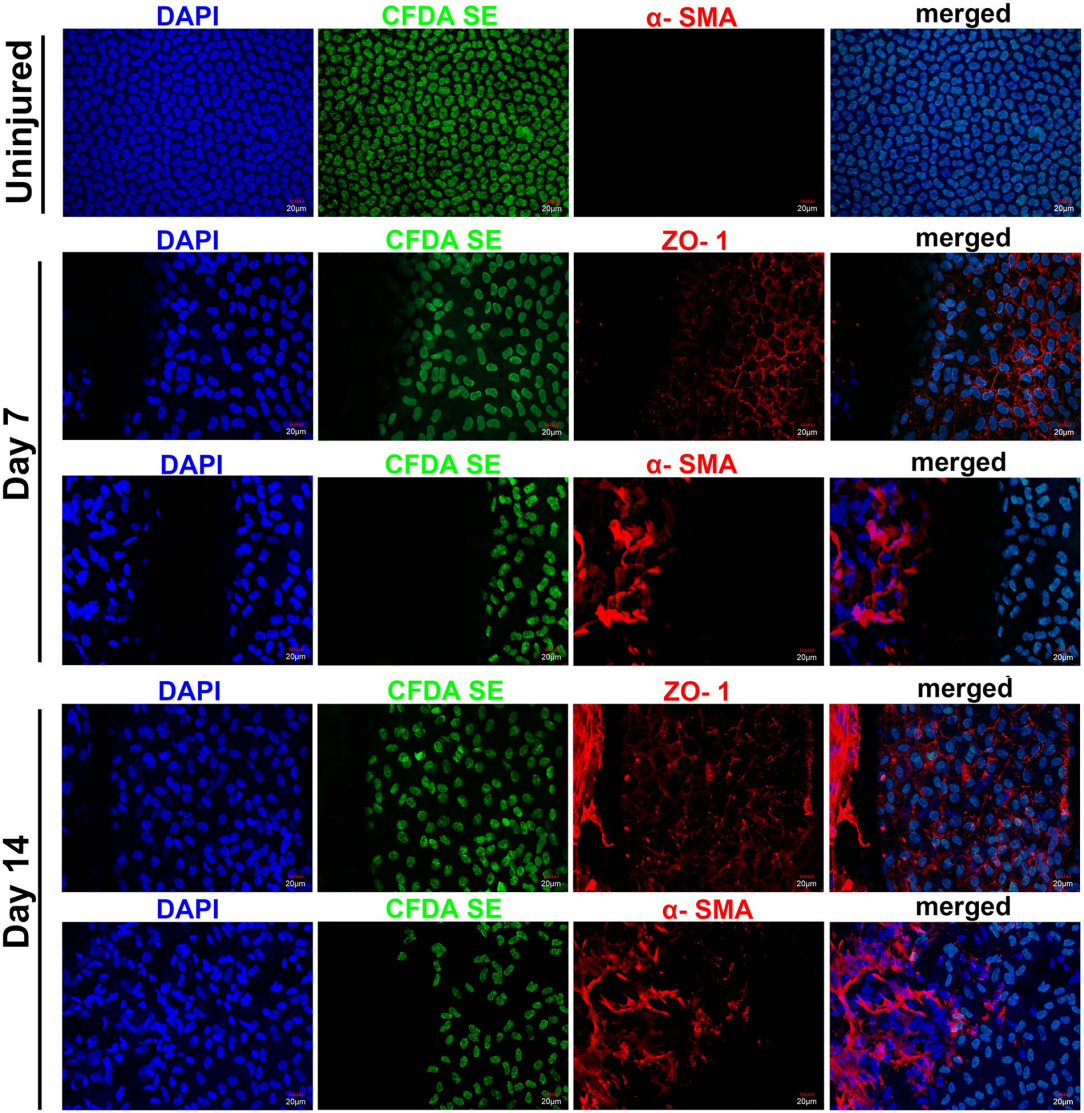
Whole-mount staining of ZO-1 and α-SMA on a DM stripping cornea. ZO-1 expression in the endothelial cells close to the wound border decreased on day 7, cells on the stromal surface of the wound area were not labeled with CFDA SE but were highly expressed with α-SMA. On day 14, ZO-1 expression was regained in the endothelial cells close to the wound border. CFDA SE negative cells close to the wound border also showed positive ZO-1 expression. α-SMA was maintained in the CFDA SE negative cells, while it was negative in the CFDA SE-labeled cells. Bars represent 20 μm.

In the DM stripping rabbits, ZO-1 expression in the endothelial cells close to the wound border also decreased on day 7, and cells on the stromal surface of the wound area were not labelled with CFDA SE, although highly expressed with α-SMA, further confirmed these α-SMA positive cells were not originated from pre-labelled corneal endothelial cells (Fig. 8). On day 14, ZO-1 expression was regained in the endothelial cells close to the wound border, to the extent of weaker than normal control. CFDA SE negative cells close to the wound border also showed positive ZO-1 expression, while it was not as organized as was demonstrated in the normal endothelial cells. The α-SMA was maintained in the CFDA SE negative cells, while it was negative in the CFDA SE-labelled endothelial cells on day 7 and day 14 (Fig. 8).

**Figure 9:**
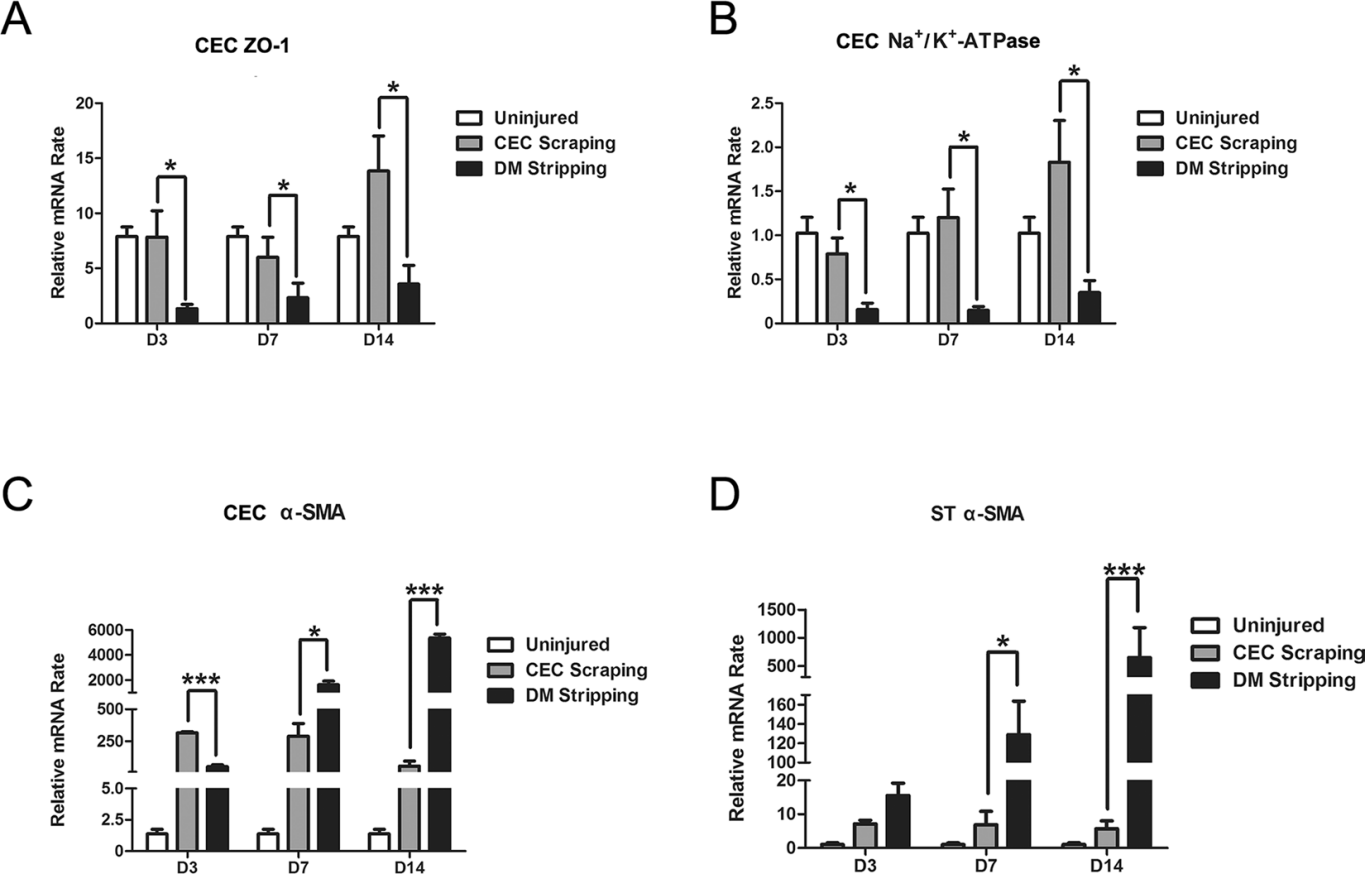
Gene expression in corneal endothelial cells and stromal cells after endothelial injury. ZO-1 gene expression in CECs after scraping injury was significantly higher than that in CECs after DM stripping at day 3, 7, and 14 (**A**). Na^+^/K^+^ ATPase gene expression significantly decreased after DM stripping at day 3, 7, and 14 (**B**). α-SMA gene expression in CECs dramatically increased after scraping injury and DM stripping at day 3, 7, and 14. α-SMA expression was higher in CEC scraping group at day 3, while was higher in DM stripping group at day 7 and day 14 (**C**). α-SMA gene expression in central corneal stroma showed mild increase in CEC scraping group at day 3, 7, and 14. While there was dramatic increase in DM stripping group at day 7 and day 14 (**D**). (*p < 0.05; **p < 0.01; ***p < 0.001).

To further illustrate the phenotypic change of the endothelial cells during wound healing process after CEC scraping or DM stripping injury, we performed quantitative real-time PCR on the corneal endothelial cells and stromal cells after endothelial injury. In CEC scraping group, ZO-1 expression mildly decreased at day 7 and increased at day 14 to a level even higher than that of normal control. However, ZO-1 expression dramatically decreased at day 3 and maintained in low level day at 7 and 14 in DM stripping group, and was significantly lower than that of CEC scraping group (Fig. 9A). Sodium-potassium adenosine triphosphatase (Na^+^/K^+^-ATPase) gene expression showed similar pattern as ZO-1, and it is lower in DM stripping group at day 3, 7, and 14, compared with CEC scraping group (Fig. 9B). α-SMA gene expression in CECs dramatically increased after scraping injury and DM stripping at day 3, 7, and 14. It was higher in CEC scraping group at day 3, while was higher in DM stripping group at day 7 and day 14 (Fig. 9C). We also detected α-SMA gene expression in central corneal stromal cells, and found that there was only mild increase in CEC scraping group at day 3, 7, and 14. However, α-SMA expression was dramatically increased in DM stripping group at all the time points, and it was higher than that of CEC scraping group at day 7 and 14 (Fig. 9D), indicating activation of stromal cells after DM stripping.

## Discussion

Our study demonstrated the prognosis difference between a CEC scraping injury and a DM stripping injury to the corneal endothelium of rabbits. Previous report has shown that a scraping injury to the corneal endothelial cells around 1 × 10^5^ μm^2^ in rabbits could completely heal without any scar formation within 48 hours^12^. In the current study, a 5 mm diameter denuded DM surface with wound area of approximately 2 × 10^7^ μm^2^ could be repopulated by corneal endothelial cells within 14 days after the scraping injury. During the wound healing process with respect to the scrape injuries, the tight junction of endothelial cells, close to the forefront area, was released, and these cells acquired a myofibroblast phenotype, which was confirmed by α-SMA expression. After the endothelial wound closure at 14 days post-injury, the endothelial tight junction was restored; meanwhile, α-SMA staining became negative, indicating that the cells reversed to an endothelial phenotype. Therefore, with the presence of intact DM, rabbit corneal endothelial cells surrounding the wound area showed EMT first, and exhibited mesenchymal-endothelial transition (MET) after the wound closure. This is the first study in which sequential EMT and MET procedures during corneal endothelial wound healing have been demonstrated.

It is interesting to note that when we used a stripping injury method to remove corneal endothelial cells and DM simultaneously, there was retrocorneal fibrous membrane formation in all the animals 14 days after the injury. Cell tracing showed that there were very few endothelial cells that migrated across the stripping line into the wound area, and the α-SMA positive cells in the wound area did not show CFDA SE labeling, indicating that the cells in the fibrous membrane did not originate from endothelial cells. As we know, the compact DM separates the corneal stroma and the endothelium; after DM removal, the underneath stroma was exposed to the aqueous humor, which contains growth factors such as TGF-β family members^35^. It is possible that keratocytes close to the wound surface were activated by TGF-β and differentiated into α-SMA-positive myofibroblasts; such an effect of TGF-β on keratocyte has been well studied previously^36, 37^. Previous study using transcorneal freeze (TCF) injury in rabbit eye also showed development of retrocorneal fibrous membrane. However, there was fibrin clot overlying the wound after TCF injury, and there were inflammatory cells infiltrated in the posterior corneal stroma^10^. In the current DM stripping model, there was no fibrin clot formation and inflammatory cell infiltration after injury, therefore the cell source of the fibrous membrane may be different from that of TCF injury. Even though, we still could not rule out the possibility that circulating cells from the aqueous humor might also contribute to the fibrous membrane formation in certain degree, further study is needed to answer this question.

In the stripping injury cornea, although there was fibrous membrane formation in the central cornea, we found the corneal edema was actually recovered under the slit-lamp microscope and that the anterior part of the central corneal stroma, as well as the peripheral corneal stroma, was clear 14 days after the wound was administered. This result indicated that the corneal pump function was recovered. Confocal images showed that the cells on the posterior surface of the wound area exhibited an endothelial-like shape. Immunostaining further confirmed that these cells simultaneously expressed α-SMA and ZO-1. This result supports the notion that α-SMA-positive cells may partially acquire an endothelial phenotype. Since keratocytes and corneal endothelial cells both originate from the neural crest, it raises the possibility that keratocytes may transdifferentiate into corneal endothelial-like cells during the wound healing process.

**Figure 10:**
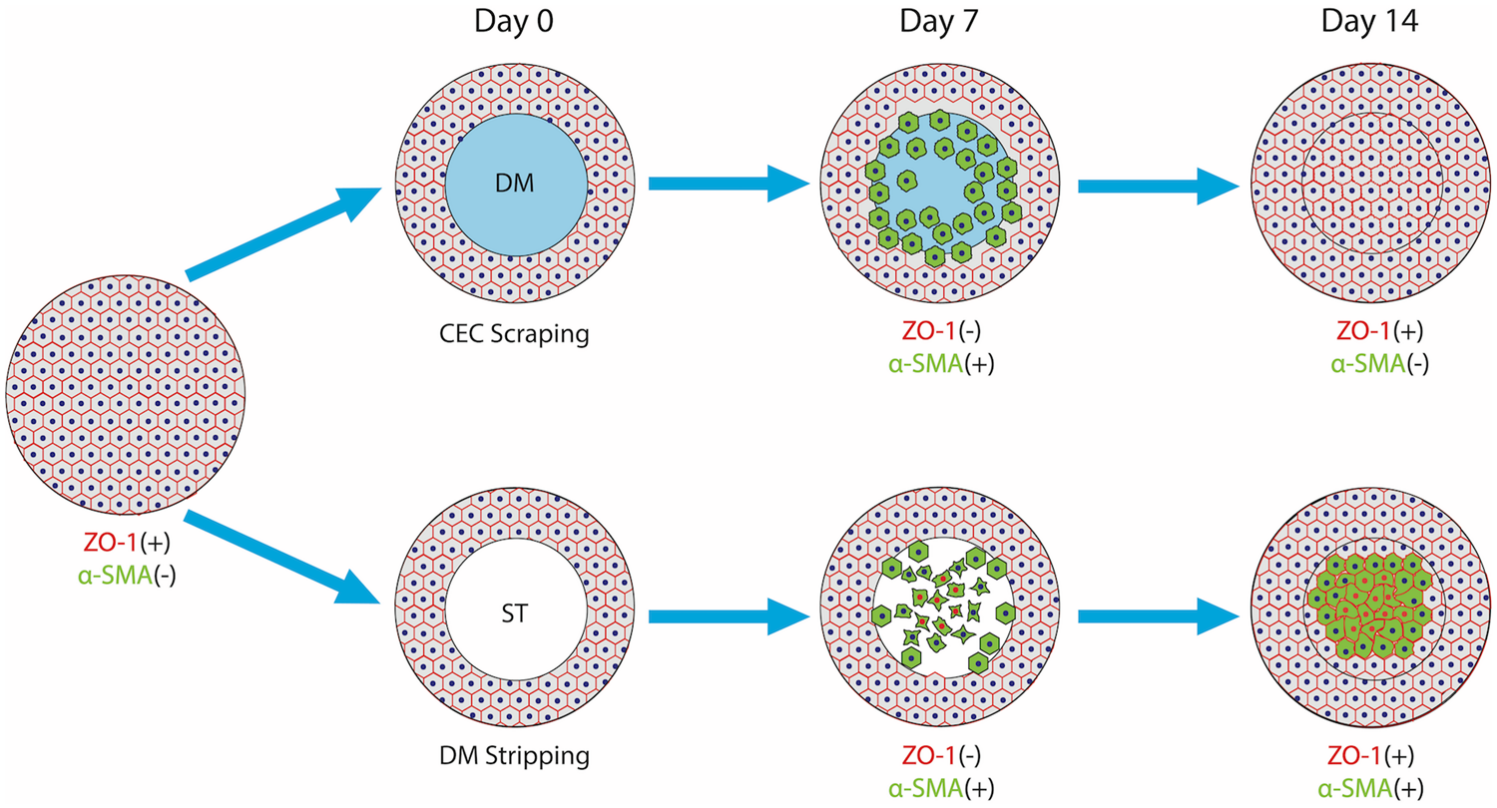
Schematic drawing on the hypothesis of corneal endothelial would healing process after CEC scraping or DM stripping injury. After CEC scraping injury, central corneal endothelial cells were removed and the underneath Descemet’s membrane (DM) was exposed at day 0. Seven days after the injury, CECs in the peripheral cornea migrated into the wounding area. These cells exhibited endothelial mesenchymal transition. At day 14, the wounding area was resurfaced by the migrating cells, and these cells regained endothelial phenotype through mesenchymal endothelial transition. After DM stripping injury, central corneal endothelial cells were also removed and the central corneal stroma (ST) was exposed at day 0. Seven days after the injury, few CECs in the peripheral cornea migrated into the wounding area. On the other hand, some keratocytes were activated and changed into myofibroblastic phenotype (red nuclei cells). At day 14, the wounding area was resurfaced by two types of cells, one from migrated endothelial cells, while the other from keratocytes. These cells partially regained endothelial cell phenotype.

Based on these findings, we can conclude that endothelial healing after a scrape injury mimics the tissue regeneration process; however, healing after a stripping injury is more similar to a fibrotic wound closure procedure. We summarized this hypothesis in a schematic drawing (Fig. 10). Regarding the mechanism, we presume that DM may provide a better scaffold for corneal endothelial migration and proliferation. The DM is made up of a series of two-dimensional grids, each consisting of nodes and connecting internodal filaments^38^. The DM showed a higher stress attainment and stiffness property in both lateral and axial directions at moderate strain values when compared with the lens capsule^34^. Meanwhile, a mechanical properties study showed that the elastic modulus of the DM is much higher than that of the anterior basement membrane of the corneal epithelium^39^. These features of the DM could maintain its original area and radian to prevent corneal deformation in the early stages of CEC wounds, thereby relieving corneal edema, cell damage, and related inflammation.

On the other hand, the DM could prevent the infiltration of certain proteins from the aqueous humor into the corneal stroma. The average pore size of the DM is about 38 nm, less than the 92 nm average pore size of the anterior basement membrane^39^. It is therefore likely that the DM could prevent infiltration of big molecules, such as the TGF-β, in the aqueous humor and into the corneal stroma, thus serving the function of preventing stromal fibrosis. This may be the reason that there was no obvious keratocyte activation and abnormal differentiation in the CEC scraping injuries.

Although the rabbit corneal endothelium and the human corneal endothelium are significantly different with respect to cell proliferation, our study is clinically relevant. Endothelial transplantation surgeries, such as Descemet’s membrane endothelial keratoplasty (DMEK), Descemet’s stripping endothelial keratoplasty (DSEK), and Descemet’s stripping automated endothelial keratoplasty (DSAEK), are frequently performed in the treatment of corneal endothelial diseases^40^, and the DM was normally stripped away, with a damaged endothelium, in these surgeries^41^. Long-term follow-up showed a high incidence of posterior stromal and graft interface haze^42, 43^. Aside from this, the retrocorneal membrane can develop postoperatively in cases with dislocation or detachment of Descemet’s membrane endothelial graft^44^. We presume that these complications are connected to the DM removal and the exposure of the corneal stroma to the aqueous humor. Recent studies on the clinical outcomes of non-Descemet stripping automated endothelial keratoplasty found no interface haze if the DM was preserved in the surgery^45, 46^, supporting our notion that corneal homeostasis could be better maintained after injury or surgery if the DM is maintained intact.

In conclusion, our study successfully simulated the CEC wound healing process after surgical intervention in a rabbit model and demonstrated the important role of the DM in corneal endothelial regeneration in rabbits. This study may shed new light on the suggestion that DM affects the corneal endothelial wound repair in humans. It also provided insights for future study on DM based corneal endothelial tissue engineering.

## Methods

### Experimental animals

A total of 36 yellow rabbits (weighing 2.5–3.0 kg) were used in the study. They were randomly assigned to 2 groups of 18 rabbits each: the Descemet’s membrane (DM) stripping group and the corneal endothelial cell (CEC) scraping group. The animals were housed in individual cages at a constant room temperature (19–23 °C), humidity of 30% to 50%, and a constant 12-hour light-dark cycle. Food and water were provided at libitum. All animals were handled according to the ARVO Statement for the Use of Animals in Ophthalmic and Vision Research, and the study received the approval of the Animal Ethical Committee of Xiamen University.

### Corneal endothelium surgery

The rabbits were anesthetized via an intramuscular injection of ketamine hydrochloride (14 mg/kg) and xylazine hydrochloride (7 mg/kg), and then topically anesthetized with proxymetacaine hydrochloride (Alcon, Fort Worth, TX, US). One eye of each animal was subjected to an endothelial injury. Experiment eyes were treated with a drop of 5% tropicamide eyedrop (Santen, Osaka, Japan) 3 times to enlarge the pupil. When anesthesia was achieved, a lid speculum was inserted, and the epithelial surface of the central cornea was marked with a 5 mm trephine. A penetrated corneal incision was made with a 2.6 mm slit knife through the peripheral cornea at temporal side. After that, 0.1 mL heparin (12500 U, 2 mL, Wanban Biomedical Pharmaceuticals Co. Ltd, Jiangsu, China) and 0.2 mL Healon (14 mg/mL Wanban Biomedical Pharmaceuticals Co. Ltd, Shanghai, China) were injected into the anterior chamber sequentially to reduce exudation and to prop up the anterior chamber. For the DM stripping injury, the DM in the surgical site was torn with micro-forceps along the imprinted edge to remove the DM, as well as the endothelial cells. For the CEC scraping injury, the corneal endothelial cells in the surgical site of a 5 mm diameter were gently scraped with a cell scraper to avoid DM damage. At the end of the surgery, the anterior chamber was irrigated with a Hanks-based saline solution to remove the Healon and endothelial cell debris. All operations were performed by the same surgeon (WL) and one aide. Norfloxacin eye drops (WuJing Pharmaceutical Company, Wuhan, China) were applied to the surgical eyes 3 times per day for 3 days post-surgery.

### Corneal endothelium intravital labeling

In order to trace the corneal endothelial cells, the corneal endothelium was intravitally labelled with CFDA SE Cell Tracer (Vybrant® CFDA SE Cell Tracer Kit, Invitrogen, Carlsbad, CA, US). To do this, 6 rabbits were anesthetized as described above. Anterior chamber paracentesis was performed on the peripheral cornea at temporal side; 0.2 mL aqueous humor was then exchanged by CFDA SE Cell Tracer using a 1 mL syringe. DM stripping or CEC scraping injury was performed 24 hours after intravital labelling.

### General post-surgery observation

After DM stripping or CEC scraping injury, slit-lamp microscopy was performed daily on the rabbit eyes to observe morphological changes of the cornea and inflammatory reactions of the anterior chambers, corneas, and ocular surfaces; images were obtained on postoperative days 0, 3, 7, and 14. Ultrasonic A pachymeter (UP-1000, Nidek, Gamagori, Japan) was used to measure corneal thickness on days 0, 3, 7, and 14 post-injury. Five points on the surgical site and another five points on the peripheral cornea external to the surgical site were measured, respectively. Six animals from each group were investigated for post-injury observation.

### *In vitro* confocal microscopy

Rabbits in both groups were sacrificed at different points of time on days 3, 7, and 14, with 3 animals at each time point in each group. The eyes were enucleated immediately after the sacrifice and scanned with a Heidelberg Retina Tomograph III Rostock Corneal Module HRT III RCM (Heidelberg Engineering GmbH, Dossenheim, Germany). The corneas were scanned in 3 different areas: the surgical site, the surgical boundary, and the peripheral cornea. At least 30 non-overlapping images were obtained from each layer of the corneal epithelium and from the superficial and deeper stroma, as well as from the corneal endothelium.

### Alizarin red S staining

After sacrifice of the animals at different time points, the corneal tissues were removed from the eyeball for section staining or whole-mount staining. To determine the endothelial damage after different surgeries, normal cornea and corneal tissues harvested right after the surgeries were placed endothelial side up and Alizarin red S (0.2%; pH 4.2; Sigma, St. Louis, MO, US) was applied to the endothelium for 5 min. After that the corneal tissues were rinsed with PBS for 5 min followed by fixation with 4% paraformaldehyde for 20 min at room temperature, after additional rinse with PBS for 5 min, the corneas were cut into 4 pieces and mounted on a glass slide, the corneal endothelium was observed and photographed with a Leica upright microscope (DM2500, Leica Microsystems, Wetzlar, Germany).

### Immunofluorescence staining

Rabbit corneal tissues for section staining were embedded with optimal cutting temperature (OCT) compound and frozen at −80 °C. For immunofluorescence staining, cryosections of 6 μm thickness were fixed in acetone at −20 °C for 15 minutes and incubated in 0.2% Triton X-100 for 20 minutes. After 3 rinses with PBS for 5 minutes each and preincubation with 2% bovine serum albumin (BSA) for 1 hour at room temperature, sections were incubated with α-SMA antibody (1:200, ab18147, Abcam, Cambridge, MA, US) at 4 °C overnight. After 3 washes with PBS for 15 minutes, the sections were incubated with Texas Red 66127-conjugated IgG (1:50; Jakson, ImmuResearch, Langcaster, PA, US) for 1 hour. After 3 additional PBS washes for 5 minutes each, the sections were mounted with an anti-fade solution containing DAPI (Vector, Burlingame, CA, US).

Tissues for whole-mount immunofluorescence staining were fixed in 4% paraformaldehyde at 4 °C for 1 hour and incubated in 0.2% Triton X-100 for 1 hour. After 3 rinses with PBS for 30 minutes each, the tissues were incubated with 2% BSA for 8 hours at room temperature and incubated with α-SMA antibody (1:200) and ZO-1 antibody (1:200, 61-7300, Zymed, San Diego, CA, US) separately at 4 °C overnight. After 3 washes with PBS for 30 minutes each, the tissues were incubated with Texas Red 66127-conjugated IgG (1:50; Jakson, ImmuResearch, Langcaster, PA, US) for 1 hour. After 3 additional PBS washes for 5 minutes each, the tissues were counterstained with an anti-fade solution containing DAPI (Vector, Burlingame, CA, US). All the sections and tissues were photographed using a confocal laser scanning microscope (Fluoview FV1000; Olympus, Tokyo, Japan).

### RNA isolation and quantitative real-time RT-PCR analysis

After scarification of the animals at different time points, corneal epithelial cells were removed using an Algerbrush II rotating burr (Alger Equipment Co., Inc., Lago Vista, TX). After that, the central cornea was cut with 10-mm trephine, total RNA was then extracted from rabbit corneal endothelium and stromal tissues separately with the use of TRIzol (Invitrogen, Carlsbad, CA) and was reverse-transcribed to cDNA by the ExScript RT Reagent kit (Takara, Dalian, China) according to the manufacturer’s protocol. Real-time PCR was performed using a SYBR Premix Ex Taq Kit (RR420A; TaKaRa) using a StepOne plus Real-Time PCR System (Applied Biosystems, Darmstadt, Germany). The rabbit gene primers were designed using Primer 3 system and their sequences are showed as follows: β-actin, 5′-CGGCTACAAAGACGGCAAAC-3′, 5′-GAACAGGCAGCACATTTGGG-3′; ZO-1, 5′-AGTTTGGCAGCAAGAGATGG-3′, 5′-GCTGTCAGAAAGGTCAGGGA-3′; α-SMA, 5′-GGTGGGAATGGGGCAAAAAG-3′, 5′-TGGATGTTCTTCAGGGGCAA-3′; sodium-potassium adenosine triphosphatase (Na^+^/K^+^-ATPase) β1, 5′-CGGCTACAAAGACGGCAAAC-3′, 5′-GAACAGGCAGCACATTTGGG-3′. The amplification program was comprised of an initial denaturation step at 95 °C for 10 minutes, followed by denaturation step at 95 °C for 10 seconds and annealing and extension at 60 °C for 30 seconds for 40 cycles. Melting curve analysis was conducted at once by raising the temperature from 65 °C to 95 °C. SYBR Green fluorescence was measured after each extension step, and the specificity of amplification was evaluated by melting curve analysis.

### Statistical analysis

Summary data are reported as means ± S.D. In the comparison of corneal stoma thickness, two-factor repetitive measurement was used for the overall comparison. LSD-*t* test was used for intergroup comparison in each time point, paired *t*-test was used for the intra-group comparison of different time points. A value of *p* < 0.05 was considered statistically significant.
